# *Inflamm-Aging* Is Associated with Lower Plasma PTX3 Concentrations and an Impaired Capacity of PBMCs to Express hTERT following LPS Stimulation

**DOI:** 10.1155/2019/2324193

**Published:** 2019-09-12

**Authors:** Aaron L. Slusher, Tiffany M. Zúñiga, Edmund O. Acevedo

**Affiliations:** ^1^Department of Kinesiology and Health Sciences, Virginia Commonwealth University, Richmond, VA, USA; ^2^School of Kinesiology, University of Michigan, Ann Arbor, MI, USA; ^3^Department of Nutritional Sciences, University of Arizona, Tucson, AZ, USA

## Abstract

Age-related elevations in proinflammatory cytokines, known as *inflamm-aging*, are associated with shorter immune cell telomere lengths. *Purpose*. This study examined the relationship of plasma PTX3 concentrations, a biomarker of appropriate immune function, with telomere length in 15 middle-aged (40-64 years) and 15 young adults (20-31 years). In addition, PBMCs were isolated from middle-aged and young adults to examine their capacity to express a key mechanistic component of telomere length maintenance, human telomerase reverse transcriptase (hTERT), following *ex vivo* cellular stimulation. *Methods*. Plasma PTX3 and inflammatory cytokines (i.e., IL-6, IL-10, TGF-*β*, and TNF-*α*), PBMC telomere lengths, and PBMC hTERT gene expression and inflammatory protein secretion following exposure to LPS, PTX3, and PTX3+LPS were measured. *Results*. Aging was accompanied by the accumulation of centrally located visceral adipose tissue, without changes in body weight and BMI, and alterations in the systemic inflammatory milieu (decreased plasma PTX3 and TGF-*β*; increased TNF-*α* (*p* ≤ 0.050)). In addition, shorter telomere lengths in middle-aged compared to young adults (*p* = 0.011) were negatively associated with age, body fat percentages, and plasma TNF-*α* (*r* = −0.404, *p* = 0.027; *r* = −0.427, *p* = 0.019; and *r* = −0.323, *p* = 0.041, respectively). Finally, the capacity of PBMCs to increase hTERT gene expression following *ex vivo* stimulation was impaired in middle-aged compared to young adults (*p* = 0.033) and negatively associated with telomere lengths (*r* = 0.353, *p* = 0.028). *Conclusions*. Proinflammation and the impaired hTERT gene expression capacity of PBMCs may contribute to age-related telomere attrition and disease.

## 1. Introduction

Aging is accompanied by the chronic, low-grade elevation of circulating proinflammatory cytokines (e.g., interleukin 6 (IL-6) and tumor necrosis factor alpha (TNF-*α*)) that typically manifests during middle age (40-64 years of age) [1, 2]. This phenomenon has recently been termed *inflamm-aging* [3]. The causes of *inflamm-aging* are numerous [4]. However, the pathology has been hypothesized to derive from the natural loss of subcutaneous adipose tissue (SAT) and the accumulation of centrally located visceral adipose tissue (VAT) that occurs in the absence of weight gain or changes in the body mass index (BMI) and is associated with elevated levels of circulating proinflammatory cytokines [5–7]. Furthermore, VAT expresses and produces greater amounts of proinflammatory cytokines with increased age [8], and within the stromal vascular fraction of VAT, the number of T cell lymphocytes increases and resident monocyte-derived macrophages are polarized towards an M1, proinflammatory, phenotype [9]. As a result, the increased cellular production and secretion of proinflammatory cytokines into circulation (e.g., IL-6 and TNF-*α*), in combination with the decreased concentration of anti-inflammatory cytokines (e.g., IL-10 and transforming growth factor beta (TFG-*β*)), contribute to the elevated risk of premature morbidity and mortality from age-related diseases, including cardiovascular disease (CVD) and metabolic dysregulation [10, 11].

Telomeres maintain the “youthful” function of immune cells, and shortened telomere lengths are considered to be a biological marker of cellular aging [12]. More specifically, telomeres are formed by thousands of hexameric 5′(TTAGGG)_n_3′ repeats located at the ends of linear chromosomes and protect chromosomes from degradation and end-to-end fusion [13, 14]. Although leukocyte telomere lengths shorten as a natural consequence of aging [15, 16], the persistent exposure of circulating immune cells (i.e., T cell lymphocytes and monocytes) to age-related proinflammatory profiles may accelerate telomere attrition rates [17–20]. Telomere length predicts the replicative capacity of dividing cells [21], and once telomeres reach a critically shortened length, cells enter an irreversible state of replication-induced cellular senescence [22, 23]. Consequently, aged cells, including leukocytes, exhibit a senescent-associated secretory phenotype (SASP) that further elevates levels of proinflammatory cytokines [19, 24, 25]. More worrisome, cellular senescence spreads from cell to cell and other organ systems, including adipose tissue [26, 27], exacerbating the progression of age-related proinflammatory diseases [19, 25, 28, 29].

Prior to senescence, leukocytes express human telomerase reverse transcriptase (hTERT). hTERT is the rate-limiting component and surrogate marker of the telomerase enzyme that is recruited to facilitate the generation of new telomeric DNA that maintains the length and structural integrity of telomeres [30–32]. In response to acute inflammatory challenge, hTERT gene expression is significantly increased in THP-1 macrophage cells following stimulation with LPS and other proinflammatory stimulants [33]. Interestingly, basic research in the human T cell clones costimulated with anti-CD3 and anti-CD28 antibodies demonstrates that the capacity of activated T cells to transiently express hTERT decreases as cells approach their replicative capacity (early vs. late population doubling; [34]). These findings suggest that the progressive and persistent inflammatory assault observed with *inflamm-aging* may also impair the capacity of leukocytes to express the hTERT gene in middle-aged compared to young adults. Consequently, the decreased capacity of leukocytes to express the hTERT gene has been shown to be a central factor associated with telomere length shortening and the induction of cellular senescence [33, 35]. However, the hypotheses that age-related changes in adiposity, independent of changes in body weight and BMI, and elevations of proinflammatory cytokines alter the length of telomeres and associated mechanisms (i.e., hTERT gene expression) have yet to be thoroughly investigated in healthy human adults. Such gaps within the literature highlight the need to examine hTERT gene expression capacity as a potential cellular target which links the mechanistic consequences of *inflamm-aging* to telomere length-dependent replication-induced cellular senescence.

Pentraxin 3 (PTX3) is a counterregulatory protein that is expressed and secreted from isolated leukocytes in concert with various inflammatory proteins (e.g., IL-6, IL-10, TGF-*β*, and TNF-*α*) following LPS stimulation [36–39]. Elevated PTX3 production is required to prevent overactivation of the inflammatory signaling pathway, and increased plasma PTX3 concentrations are considered an indicator of appropriate immune function in young, healthy adults [39–43]. In addition, Pavanello et al. [44] have recently demonstrated that elevated concentrations of plasma PTX3 are associated with longer telomere lengths in healthy middle-aged adults. However, whether or not plasma PTX3 concentrations are altered as a consequence of the *inflamm-aging* phenotype that impairs telomeric-associated mechanisms remains unknown. Therefore, age-related changes in plasma PTX3 concentrations and the relationship with telomere lengths were examined in middle-aged (40-64 years of age) and young adults (20-31 years of age). The capacity of PTX3 to modulate the LPS-induced inflammatory response and hTERT gene expression in PBMCs isolated from middle-aged and young adults was also examined.

## 2. Materials and Methods

### 2.1. Research Participants

A total of thirty healthy young (*n* = 15; between 20 and 31 years of age) and middle-aged (*n* = 15; between 40 and 64 years of age) adults were recruited to participate in this study. All subjects presented with a BMI associated with a reduced risk of CVD according to Stevens et al. [45]. Prior to their enrollment, each subject provided their informed consent and completed a medical history questionnaire to verify that they had not been previously diagnosed with any cardiovascular, metabolic, renal, liver, pulmonary, asthmatic, rheumatic, or other inflammatory disease/condition, were not currently under the administration of medication known to alter their inflammatory or metabolic profiles, or within the past 10 years had not been diagnosed with any cancer requiring radiation or chemotherapy treatment. Furthermore, subjects who were currently using or have used tobacco products within the past six months or who consumed >10 alcoholic beverages per week on average were excluded from participation in the study. Finally, all subjects completed a 7-day International Physical Activity Questionnaire to verify that they participated in ≤150 minutes of moderate to vigorous physical activity per week [46] and were therefore classified as physically inactive according to the American College of Sports Medicine [47]. The University's Institutional Review Board approved the study.

### 2.2. Laboratory Procedure

Subjects arrived at the laboratory between 6 : 30 and 8 : 30 o'clock in the morning following an overnight fast of at least eight hours. In addition, each subject abstained from alcohol, caffeine intake, and moderate-to-vigorous physical activity for at least 24 hours prior to their participation. Immediately upon arrival, anthropometric measures were obtained, including an assessment of height and weight to determine the BMI in kilograms per meters squared (kg/m^2^), waist and hip circumferences to determine the W : H ratio, BF% evaluated by air displacement plethysmography from the measured lung volume using the BOD POD (COSMED; Chicago, IL, USA), and sagittal diameter of the abdominal region at the level of the L4/L5 vertebrae to determine an indirect measurement of VAT [48]. Each subject was then provided a quiet resting place for at least 10 minutes to assess the resting heart rate and blood pressure.

### 2.3. Plasma PTX3 and Inflammatory Cytokine Analyses

Whole blood samples were drawn into K_2_EDTA tubes (BD Vacutainer, Franklin Lakes, NJ) from each subject's antecubital vein under quiet resting conditions. Blood samples were immediately centrifuged at 3000 RPM for 20 minutes at room temperature. Plasma supernatants were collected and stored at -80°C in cryopreservation tubes for future analysis of plasma PTX3 and TGF-*β* by standard enzyme-linked immunosorbent assay (ELISA) kits and plasma IL-6, IL-10, and TNF-*α* using high-sensitivity ELISA kits according to the manufacturer's instructions (R&D Systems, Minneapolis, MN, USA).

### 2.4. DNA Isolation and Measurement of the Relative Telomere Length

The remaining buffy coat was collected and brought to a 5 mL volume with saline, and diluted PBMCs were layered over equal volumes of Ficoll-Paque (*ρ* = 1.077 g/mL; Sigma-Aldrich, St. Louis, MO) for a 30-minute centrifugation at 400 g at room temperature. Isolated PBMCs were washed with saline three times, and pelleted cells were lysed in TRIzol (Thermo Fisher, Waltham, MA, USA) for the DNA isolation according to the manufacturer's instructions. Isolated DNA was quantified by spectrophotometry using NanoDrop 2000 (Thermo Scientific, Wilmington, DE, USA). A 15 *μ*L reaction of 2x SYBR Green Master Mix (QuantaBio, Beverly, MA, USA), nuclease-free water, target primers (Integrated DNA Technologies, Skokie IL, USA), and a 15 ng sample of total DNA were utilized to quantify the relative telomere length by assessing the ratio of telomere repeats (Tel 1b: 270 nM 5′-GGTTTTTGAGGGTGAGGGTGAGGGTGAGGGTGAGGGT-3′; Tel 2b: 900 nM 5′-TCCCGACTATCCCTATCCCTATCCCTATCCCTATCCCTA-3′) to the 36B4 reference gene (36B4u: 300 nM 5′-CAGCAAGTGGGAAGGTGTAATCC-3′; 36B4d: 500 nM 5′-CCCATTCTATCATCAACGGGTACAA-3′) (T/S ratio) using PCR methodologies in triplicate [49]. Amplification conditions for RNA detection were set to heat activation at 95°C for 2 minutes, followed by 40 cycles of denaturation at 95°C for 15 seconds and annealing at 54°C for 2 minutes. For 36B4 PCR, primers were incubated as above, followed by 40 cycles of denaturation at 95°C for 15 seconds and annealing at 58°C for 1 minute. Standard curves and dissociation curves for each primer set were performed to ensure equal efficiencies and single product formation, respectively. Relative T/S ratios were calculated according to Cawthon et al. (2002). Finally, the intra-assay coefficient of variation between triplicate samples was 2.24 and 1.31% for the telomere and 36B4 gene, respectively.

### 2.5. RNA Isolation and Measurement of hTERT Gene Expression

A separate sample of isolated PBMCs was manually counted by hemocytometer and cultured in complete RPMI 1640 media supplemented with 5% fetal bovine serum and 1% penicillin and streptomycin in the presence of LPS (10 ng/mL from *E. coli* O55:B5; Sigma Aldrich, St. Louis, MO, USA), recombinant human (rh) PTX3 (100 ng/mL; R&D Systems, Minneapolis, MN, USA), or a combination of rhPTX3 and LPS (initiated by a 30-minute preincubation period with rhPTX3). Unstimulated samples served as a time course control. Stimulated cell samples were incubated at 37°C with 5% CO_2_ for a 4-hour period at a final concentration of 2 · 10^6^ cells/mL in a 6-well culture plate. Following the completion of the 4-hour culture period, PBMCs were homogenized with TRIzol (Thermo Fisher, Waltham, MA, USA) and centrifuged for 2 minutes at 16000 g in QIAshredder mini spin columns (QIAGEN, Hilden, Germany). RNA was then fully isolated using TRIzol methods according to the manufacturer's instruction and quantified by spectrophotometry using NanoDrop 2000 (Thermo Scientific, Wilmington, DE, USA). A total of 1 *μ*g of RNA was synthesized into cDNA according to the manufacturer's instructions (QuantaBio, Beverly, MA, USA), and a 15 *μ*L reaction of 2x SYBR green master mix (QuantaBio, Beverly, MA, USA), nuclease-free water, target primers (Integrated DNA Technologies, Skokie IL, USA), and a 10 ng sample of cDNA were aliquoted into a 96-well plate. Each sample was analyzed for changes in hTERT gene expression (500 nM F: 5′-TACGGCGACATGGAGAACAAG-3′; 500 nM R: 5′-GGGCATAGCTGAGGAAGGTTT-3′) against the reference gene GAPDH (200 nM F: 5′-GAAGGTGAAGGTCGGAGTC-3′; 200 nM R: 5′-GAAGATGGTGATGGGATTTC-3′) by qPCR in triplicate using a CFX96 TOUCH thermal cycler and analyzed using CFX software (Bio-Rad, Hercules, CA, USA) as previously described [50, 51]. Amplification conditions for RNA detection were set to heat activation at 95°C for 2 minutes, followed by 40 cycles of denaturation at 95°C for 15 seconds and annealing at 60°C for 30 seconds. All melting curve analyses were performed between 65°C and 95°C, and relative gene expression was calculated by 2^−ΔΔCt^ method according to Livak and Schmittgen [52].

### 2.6. Ex Vivo Stimulation of PTX3 and Inflammatory Cytokines from Isolated PBMCs

Concentrations of PTX3 and the pro- (IL-6 and TNF-*α*) and anti-inflammatory cytokines (IL-10 and TFG-*β*) secreted from isolated PBMCs acutely stimulated with LPS (10 ng/mL) were determined from cell culture supernatants. Given that inflammatory cytokines follow different secretion kinetics, isolated PBMCs were stimulated for a 4-hour culture period (described above) and, separately, a 24-hour culture period (1 · 10^6^ cells/mL) in duplicate by ELISA methods according to the manufacturer's instructions (R&D Systems, Minneapolis, MN, USA).

### 2.7. Statistical Analyses

Data analyses were performed using the Statistical Package for the Social Sciences (SPSS version 24.0). Independent *t*-tests were conducted to determine potential differences in anthropometric profiles (height, weight, BMI, waist and hip circumferences, and W : H ratio, BF%, and sagittal diameter), cardiovascular health (resting heart rate, blood pressure, and mean arterial pressure), plasma PTX3, IL-6, IL-10, TGF-*β*, and TNF-*α* concentrations, and relative telomere lengths (T/S ratio) at rest. In addition, a two-group (young adult and middle-aged adult) by four condition (time course control, LPS, PTX3, and PTX3+LPS) repeated measures analysis of variance (rmANOVA) was utilized to examine differences in hTERT gene expression levels following *ex vivo* stimulation of PBMCs following the 4-hour stimulation period in young adult and middle-aged subjects. Likewise, differences in the capacity of PBMCs to produce PTX3 and the inflammatory cytokines IL-6, IL-10, TGF-*β*, and TNF-*α* between young adult and middle-aged subjects in response to 4- and 24-hour stimulation periods were examined by a two-group by two condition (PTX3) or four condition (i.e., IL-6, IL-10, TGF-*β*, and TNF-*α*) rmANOVA. If the Mauchly's test indicated a violation of sphericity assumptions, the degrees of freedom were corrected by using Greenhouse-Geisser estimates. Finally, Pearson's correlations were utilized to examine the relationship among each variable, with application of the Benjamini-Hochberg method (false discovery rate) to correct for any limitations related to performing multiple comparisons. Statistical significance is being defined as a *p* value≤0.05.

## 3. Results

### 3.1. Subject Descriptive Characteristics

**Figure 1:**
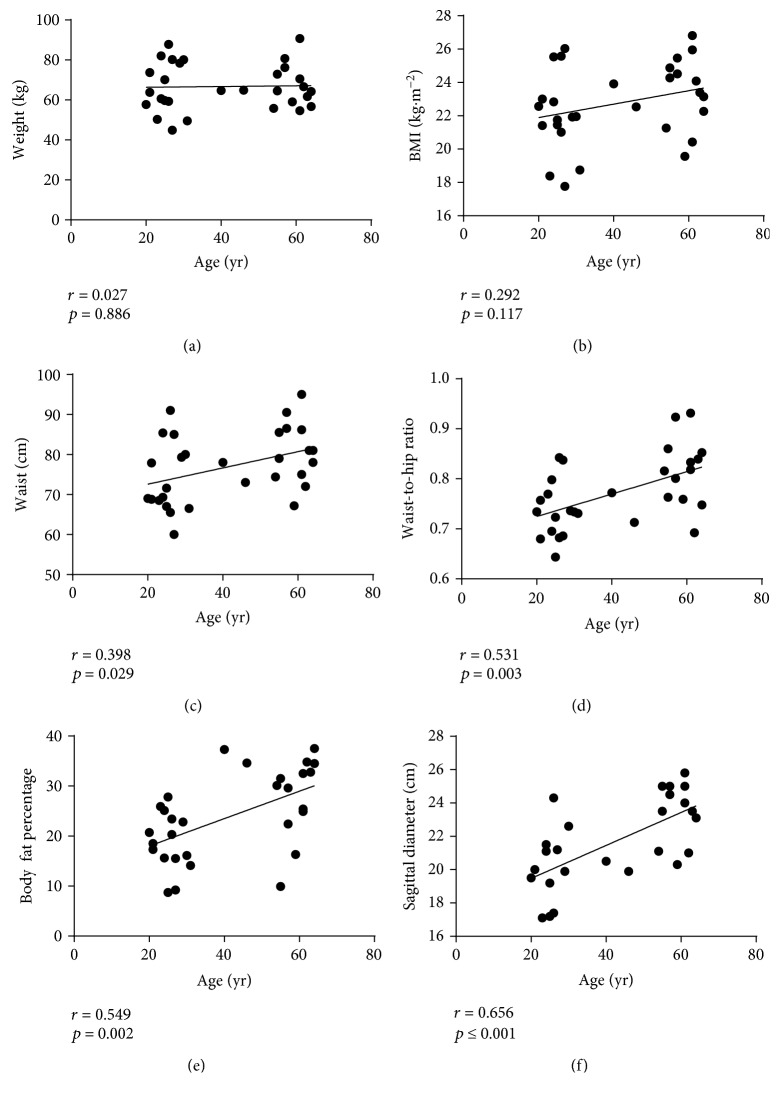
The associations of age with anthropometric characteristics in middle-aged and young adults. These data suggest that age, independent of weight gain or changes in BMI (a, b), is associated with the increased accumulation of centrally located visceral adiposity, identified by increased waist circumference, W : H ratio, body fat percentage, and sagittal diameter (c–f).

Subject characteristics are presented in Table 1. Although these data demonstrate that no differences in body weight (*t*_[28]_ = 0.087, *p* = 0.932) or BMI (*t*_[28]_ = 1.811, *p* = 0.081) were observed between middle-aged and young adults, waist circumferences (*t*_[28]_ = 2.166, *p* = 0.019), waist-to-hip (W : H) ratio (*t*_[28]_ = 3.088, *p* = 0.003), body fat percentage (BF%) (*t*_[28]_ = 4.054, *p* ≤ 0.0010), and sagittal diameter (*t*_[28]_ = 4.081, *p* ≤ 0.001) were significantly greater in middle-aged adults. Likewise, while no associations were observed between age and body weight (*r* = 0.027, *p* = 0.886) or BMI (*r* = 0.292, *p* = 0.117), age was positively associated with waist circumference (*r* = 0.398, *p* = 0.029), W : H ratio (*r* = 0.531, *p* = 0.003), BF% (*r* = 0.549, *p* = 0.002), and sagittal diameter (*r* = 0.674, *p* ≤ 0.001; Figures 1(a)–1(f)). These data support the hypothesis that natural, healthy aging in physically inactive adults is accompanied by the increased accumulation of centrally located VAT that occurs in the absence of weight gain or changes in BMI.

Furthermore, women participating in the study were shorter, weighed less, had lower BMI and smaller waist, hip circumferences, and W:H ratios, and had a greater BF% compared to men. However, no other gender differences were observed among the remaining variables examined, and therefore, analyses among men and women are not included.

### 3.2. Plasma PTX3 and Inflammatory Cytokine Concentrations

**Figure 2:**
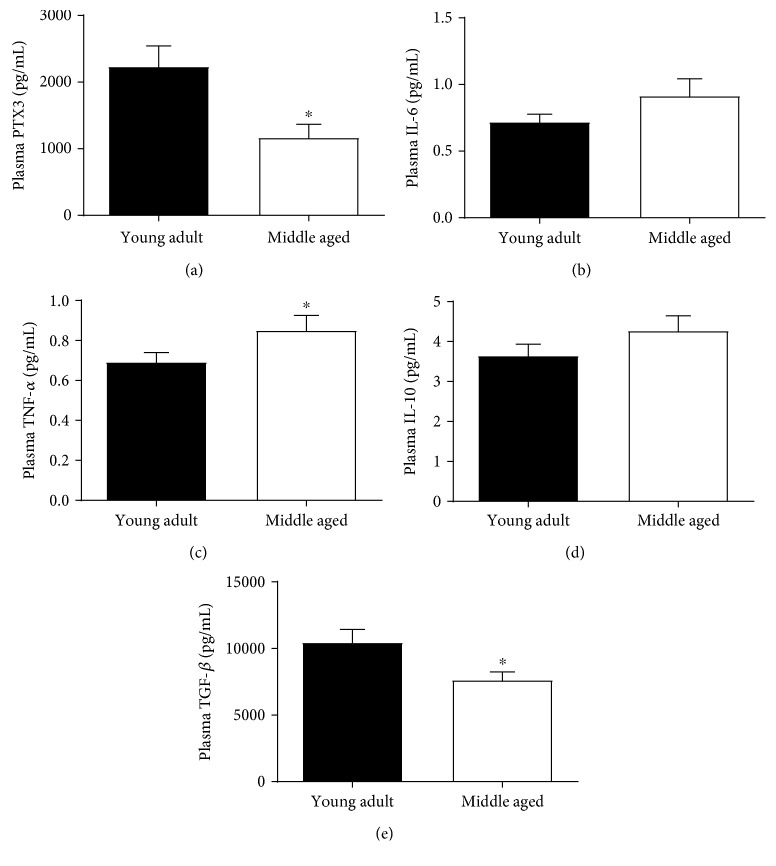
Plasma PTX3 and senescent-associated inflammatory cytokine concentrations. Plasma PTX3 concentrations were significantly lower in middle-aged compared to young adults (a). In addition, no differences in the proinflammatory cytokine IL-6 were observed (b), whereas plasma TNF-*α* concentrations were greater in middle-aged compared to young adults (c). Likewise, no differences in the anti-inflammatory cytokine IL-10 were observed (d), whereas plasma TGF-*β* concentrations were lower in middle-aged compared to young adults (e). ∗ indicates a significant difference in middle-aged compared to young adults (*p* ≤ 0.05).

Baseline concentrations of plasma PTX3 were significantly lower in middle-aged compared to young adults (*t*_[23.897]_ = 2.851, *p* = 0.009; Figure 2(a)). Although no differences in plasma IL-6 or IL-10 were observed (*t*_[28]_ = 1.384, *p* = 0.089; *t*_[28]_ = 1.303, *p* = 0.102), plasma concentrations of the proinflammatory cytokine TNF-*α* were significantly greater (*t*_[28]_ = 1.767, *p* = 0.044), and concentrations of the anti-inflammatory cytokine TGF-*β* were significantly lower in middle-aged compared to young-adults (*t*_[28]_ = 2.381, *p* = 0.012; Figures 2(b)–2(e)). No associations between plasma PTX3 concentrations and inflammatory cytokines were observed.

### 3.3. PBMC Relative Telomere Length

**Figure 3:**
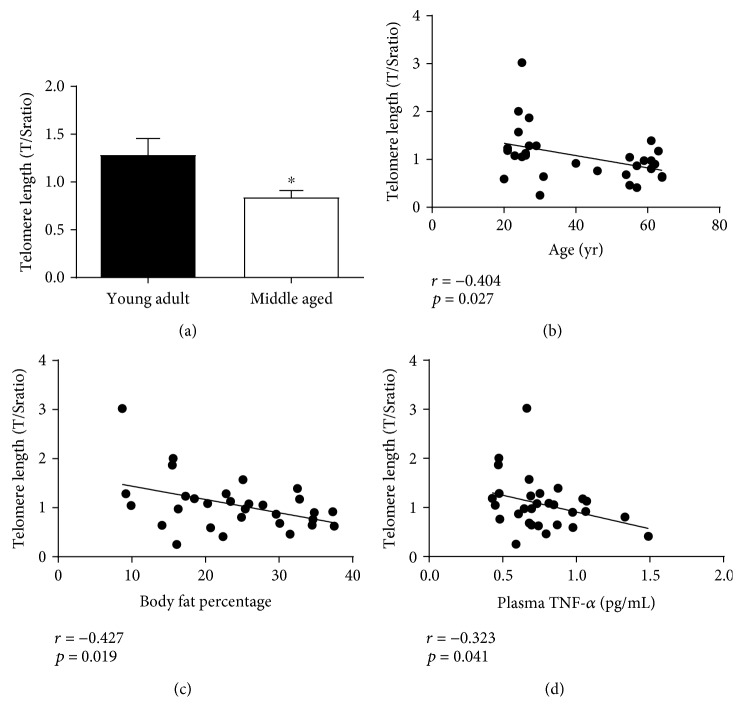
Relative telomere lengths (T/S ratio) and the associations with age and body fat percentage. Telomere lengths measured from isolated PBMCs were significantly shorter in middle-aged compared to young adults (a). In addition, telomere lengths were negatively associated with increased age, body fat percentage, and circulating concentrations of the proinflammatory cytokine TNF-*α* (b–d). However, the relationship of telomere length with age and plasma TNF-*α* concentrations were no longer significant when after controlling for differences in body fat percentage. ∗ indicates a significant difference in middle-aged compared to young adults (*p* ≤ 0.05).

Relative telomere lengths (T/S ratio) analyzed from isolated PBMCs were significantly shorter in middle-aged compared to young adults (*t*_[28]_ = 2.421, *p* = 0.011; Figure 3(a)) and negatively associated with increased age and BF% (*r* = −0.404, *p* = 0.027; *r* = −0.427, *p* = 0.019; Figures 3(b) and 3(c)). While relative telomere lengths were also negatively associated with circulating concentrations of plasma TNF-*α* (*r* = −0.323, *p* = 0.041; Figure 3(d)), no associations were observed with plasma PTX3 or other senescent-associated inflammatory cytokines. However, when controlled for differences in BF% as a function of age, the relationship between relative telomere lengths with age and plasma TNF-*α* were no longer observed (*r* = −0.225, *p* = 0.121; *r* = −0.209, *p* = 0.138).

### 3.4. PBMC hTERT Gene Expression

**Figure 4:**
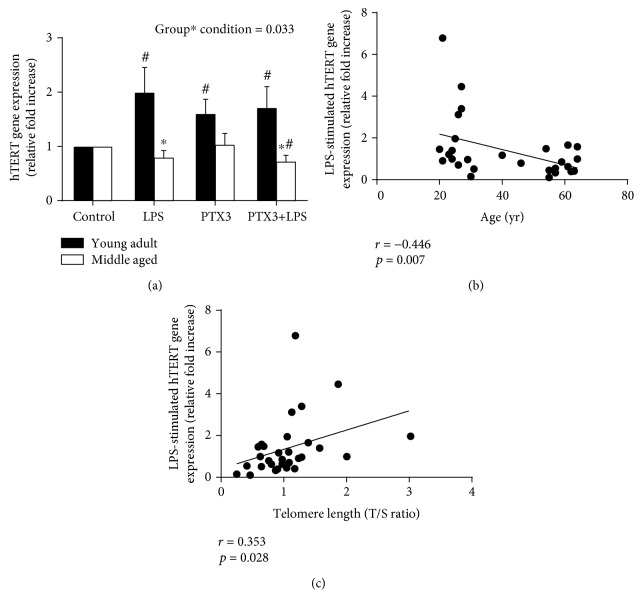
hTERT gene expression changes following 4-hour stimulation of isolated PBMCs with LPS and the associations with age and relative telomere length. hTERT gene expression was impaired in middle-aged and young adults following *ex vivo* stimulation of isolated PBMCs with LPS, PTX3, and PTX3+LPS (a). LPS-stimulated hTERT gene expression was negatively associated with age (b) and positively associated with relative telomere lengths (T/S ratio) (c). Likewise, the relationship between LPS-stimulated hTERT gene expression with age remained significant and tended toward a significant relationship with relative telomere length when controlling for differences in body fat percentage. ∗ indicates a significant difference in middle-aged compared to young adults; # indicates a significant difference compared to unstimulated control culture conditions (*p* ≤ 0.05).

In response to *ex vivo* stimulation of isolated PBMCs with LPS, PTX3, and PTX3+LPS, the capacity of middle-aged adults to express the hTERT gene relative to the time course control condition was significantly impaired under all culture conditions compared to young adults (*F*_[3, 84]_ = 3.053, *p* = 0.033; Figure 4(a)). Although PTX3 did not alter the capacity of LPS to express the hTERT gene, preincubation of PBMCs with PTX3 for 30 minutes prior to LPS stimulation was sufficient to significantly reduce hTERT gene expression relative to the time course control conditions in middle-aged adults only. Finally, changes in the LPS-stimulated hTERT gene expression (relative to the time course control condition) were negatively associated with age (*r* = −0.446, *p* = 0.007; Figure 4(b)) and positively associated with relative telomere lengths (*r* = 0.353, *p* = 0.028; Figure 4(c)). Further, the relationship in LPS-stimulated hTERT gene expression with age remained significant when controlling for BF% (*r* = −0.372, *p* = 0.024) and tended toward a significant relationship with relative telomere length (*r* = 0.275, *p* = 0.075).

### 3.5. Ex Vivo Production of PTX3 and Inflammatory Cytokines from Isolated PBMCs

**Figure 5:**
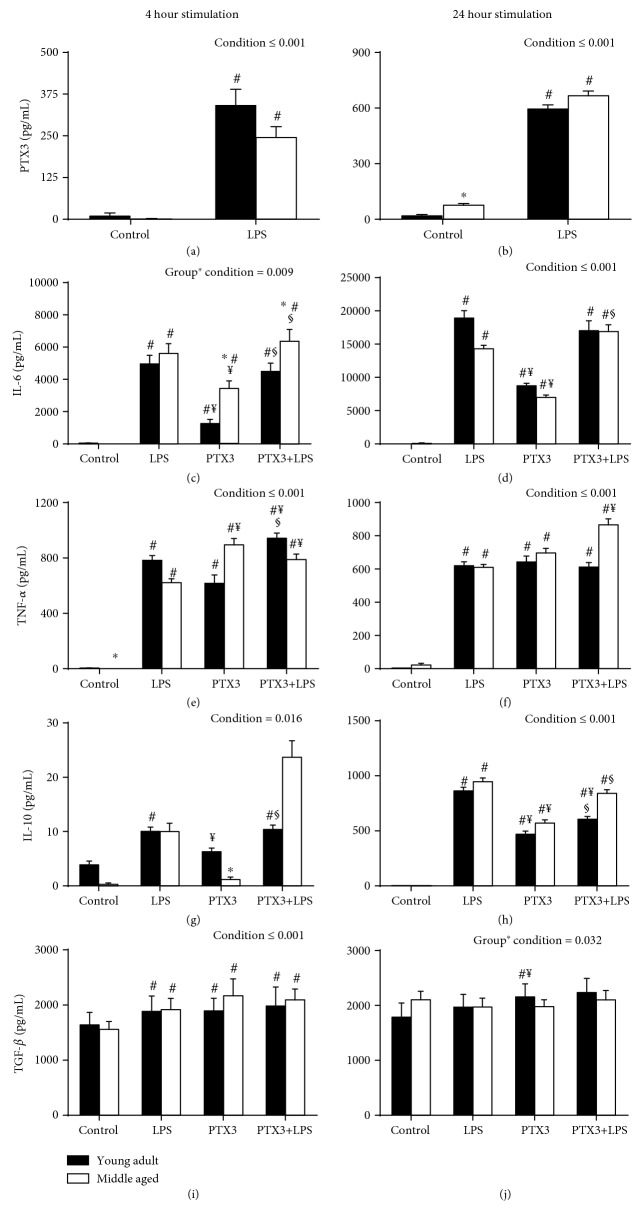
*Ex vivo* production of LPS-stimulated PTX3 (a, b) and the senescent-associated inflammatory cytokines following 4- and 24-hour stimulation of isolated PBMCs with LPS, PTX3, and PTX3+LPS in middle-aged and young adults (c–j). In addition, the capacity of PTX3 to produce and alter the LPS-stimulated production of senescent-associated inflammatory cytokines *ex vivo* was examined. ∗ indicates a significant difference in middle-aged compared to young adults; # indicates a significant difference compared to unstimulated control culture conditions; ¥ indicates a significant difference compared to LPS-stimulated culture conditions; § indicates a significant difference compared to PTX3-stimulated culture conditions (*p* ≤ 0.05).

The capacity of isolated PBMCs to produce PTX3 following 4- and 24-hour *ex vivo* stimulation with LPS was similar in middle-aged and young adults (*F*_[1, 28]_ = 116.521, *p* ≤ 0.001; *F*_[1, 28]_ = 158.38, *p* ≤ 0.001; Figures 5(a) and 5(b)). Following the 4-hour stimulation period, the overall production of IL-6 was significantly greater in middle-aged compared to young adults (*F*_[2.002, 56.043]_ = 5.094, *p* = 0.009; Figure 5(c)). However, these differences were present only in PTX3 and PTX3+LPS culture conditions. Furthermore, only a condition effect was observed for IL-6 production following the 24-hour stimulation period (*F*_[1.299, 36.358]_ = 25.91, *p* ≤ 0.001; Figure 5(d)). In addition, only a condition effect was observed for the production of TNF-*α* (*F*_[1.602, 44.849]_ = 41.682, *p* ≤ 0.001; *F*_[2.201, 61.630]_ = 57.628, *p* ≤ 0.001; Figures 5(e) and 5(f)) and IL-10 (*F*_[1.261, 35.31]_ = 5.771, *p* ≤ 0.016; *F*_[2.293, 64.211]_ = 75.961, *p* ≤ 0.001; Figures 5(g) and 5(h)) following the 4- and 24-hour stimulation periods. Finally, there was a significant increase in TGF-*β* following the 4-hour stimulation period in both groups (*F*_[2.064, 57.786]_ = 8.605, *p* ≤ 0.001; Figure 5(i)), whereas PTX3 significantly increased TGF-*β* following the 24-stimulation period in young adults only (*F*_[1.722, 48.206]_ = 3.912, *p* = 0.032; Figure 5(j)).

## 4. Discussion

This study demonstrates that natural, healthy aging in physically inactive adults is associated with the accumulation of centrally located VAT, independent of changes in body weight and BMI, and a systemic proinflammatory profile (decreased plasma PTX3 and TGF-*β*; increased plasma TNF-*α*). In addition, results from the present study suggest that the persistent exposure of immune cells to an age-related proinflammatory milieu may alter the length of telomeres by impairing the capacity of isolated PBMCs to express the hTERT gene following cellular stimulation with LPS, PTX3, and PTX3+LPS in middle-aged compared to young adults. Changes in body composition with age are typically attributed to the reduction of skeletal muscle mass, the reduced expansion of SAT, and the increased accumulation of centrally located VAT [6, 53]. In addition, alterations of adipocyte paracrine signals induce a proinflammatory phenotype of resident monocyte-derived macrophages that contribute to elevated concentrations of circulating proinflammatory cytokines [9]. This phenomenon, *inflamm-aging*, was first proposed in 2000 by Claudio Franceschi, stating that the human immune system evolved as a protective mechanism for those who had historically survived up to 40-50 years of age [3, 54]. However, life expectancy has significantly increased in recent history [55, 56]. As a result, the persistent inflammatory stimuli from an aged immune system functioning beyond its evolutionary limits may progress age-related inflammatory disease through the shortening of telomere lengths [3, 57]. In support of this hypothesis, elevated plasma concentrations of the proinflammatory cytokine TNF-*α* and decreased plasma concentrations of the anti-inflammatory cytokine TGF-*β* were observed in middle-aged compared to young adults. These findings are consistent with Álvarez-Rodríguez et al. [1], and the lack of difference in plasma IL-6 concentrations support the notion that plasma IL-6 is the “cytokine for gerontologists” with age-related differences becoming more apparent in individuals ≥ 60 years of age [1, 3, 58].

The impact of age on resting plasma PTX3 concentrations was also examined. Elevated plasma PTX3 concentrations are considered an immunological biomarker associated with the decreased risk of age-related CVD and metabolic dysfunction in otherwise healthy adults [41, 43, 59]. Similarly, although Osorio-Conles et al. [59] demonstrated that PTX3 gene expression is increased and positively associated with the presence of proinflammatory protein expression in mature adipocytes isolated from VAT, plasma PTX3 concentrations are decreased in obese compared to normal-weight individuals. Given the similarities among obesity- and age-related changes in adipose tissue [60] and the positive role that PTX3 has been shown to play in the regulation of appropriate immune function and prevention of CVD and metabolic disease [39, 41, 42, 61–63], plasma PTX3 concentrations were expectedly lower in middle-aged compared to young adults. Therefore, additional research is warranted to determine whether or not lower plasma PTX3 concentrations during middle age are related to morphological changes of VAT or an impairment of neutrophils, the primary cellular source of PTX3, to synthesize and store PTX3 throughout their maturation [64, 65].

Telomere lengths have previously been shown to be inversely associated with systemic concentrations of plasma IL-6 and TNF-*α* in elderly adults (70-79 years old; [20]) and positively associated with plasma PTX3 concentrations in adult nurses between 18 and 65 years of age [44]. Indeed, the present study indicates that age-related changes in plasma cytokine concentrations may contribute to the attrition of telomere lengths and, consequently, cellular senescence and age-related diseases that are more prevalent during the later stages of life. However, this relationship was dependent upon age-related differences in BF%, suggesting that excess adiposity with increased age underlies telomere length shortening resulting from a progressive shift towards a proinflammatory milieu. This observation indicates that an age matched comparison between normal-weight and obese individuals may be warranted in future studies. Furthermore, the lack of a relationship of plasma PTX3 with telomere lengths may be due to the physically inactive nature of the subject groups in the present study. More specifically, Pavanello et al. [44] compared PTX3 and telomere lengths in day shift and night shift nurses. Although plasma PTX3 concentrations were not different between these two groups under investigation, telomere lengths were significantly longer in night shift nurses who were also significantly younger and more physically active compared to day shift nurses. These findings suggest that physical activity may have contributed to the observed relationship of PTX3 with telomere lengths [66–68] and larger scale population studies may provide more insight into variables that influence the utilization of PTX3 as an indicator of cellular health.

Data from the present study also demonstrate that the capacity of isolated PBMCs to express the hTERT gene is impaired in healthy, middle-aged adults. Gizard et al. [33] have previously shown that LPS stimulation of human macrophages significantly increases hTERT gene expression. However, the capacity of cells to express hTERT decreases as cells approach their maximum number of replications known as the Hayflick limit [34, 69]. Ramunas et al. [35] have recently revealed that the delivery of modified mRNA encoding hTERT rapidly extends telomere lengths, increases cellular replication capacity, and, thus, prevents to onset of replication-induced cellular senescence in various human cell lines. Therefore, the inability of middle-aged adults to express hTERT following inflammatory challenge may reveal an early consequence of aging that contributes to the cascade of telomere-dependent cellular senescence disease pathology.

To examine whether or not circulating immune cells exhibit characteristics of the cell SASP, isolated PBMCs were acutely stimulated with LPS for 4- and 24-hour periods. The lack of significant differences in the PBMC-mediated inflammatory response following *ex vivo* LPS stimulation suggests that increased central adiposity, as opposed to circulating monocytes, may be the major contributor to the systemic proinflammatory milieu observed in middle-aged compared to young adults. Similarly, the lack of an elevated inflammatory response characteristic of the SASP in middle-aged compared to young adults indicates that replication-induced cellular senescence may have yet to manifest. Nonetheless, monocytes exposed to an increasingly more proinflammatory microenvironment have been shown progressively shift from a *classical* to *proinflammatory* subset in circulation and are predisposed towards an M1, proinflammatory macrophage phenotype upon their differentiation within tissue [70, 71]. Therefore, as the *inflamm-aging* phenotype progresses, circulating monocytes may exhibit an increasingly greater role in age-related disease pathology.

Shiraki et al. [40] have recently demonstrated that preincubation of macrophages and endothelial cells with 100 ng/mL of PTX3 can reduce the production of proinflammatory proteins (e.g., TNF-*α*) and increase TGF-*β*. In light of these findings, the ability of PTX3 to alter the LPS-stimulated hTERT gene expression and the production of inflammatory cytokines in PBMCs isolated from middle-aged and young adults was also examined. Interestingly, while hTERT gene expression following the preincubation of PBMCs with PTX3 was not different compared to LPS alone in middle-aged or young adults, the addition of PTX3 was sufficient to suppress hTERT gene expression relative to the time course control condition in middle-aged adults only. In addition, the preincubation of PBMCs with PTX3 did not differentially alter the LPS-stimulated production of IL-6 and TGF-*β* between subject groups. To the contrary, TNF-*α* production was enhanced in both groups following the 4-hour stimulation period, and in response to the 24-hour stimulation period, PTX3 enhanced TNF-*α* production in middle-aged adults and suppressed IL-10 production in young adults. Although these findings suggest that PTX3 may enhance the “early-” phase proinflammatory innate immune response (i.e., 4 hours), the capacity of PTX3 to alter the pro- and anti-inflammatory responses during the “late-” phase (i.e., 24 hours) innate immune response may differ with increased age. Clearly, additional research on the influence of PTX3 to regulate hTERT and other telomeric-related mechanisms responsible for maintaining telomere length across the lifespan is warranted.

## 5. Conclusions

In conclusion, telomeric biology is highly complex and tightly controlled by a variety of orchestrated and interconnected mechanisms. hTERT undoubtedly is a vital regulator of telomere maintenance and is potentially a novel cellular target that links the mechanistic consequences of *inflamm-aging* to telomere length prior to the development of telomere-dependent cellular senescence. However, hTERT only explained about 12.5% of the variance in telomere length in the present study and this relationship was dependent upon age-related differences in BF%. Likewise, the lack of telomerase activity measurement is a limitation of the present study linking hTERT gene expression capacity to telomere length attrition in healthy adults. As such, further studies should consider the impact of alternative splicing of the hTERT gene and cytoplasmic protein expression as correlated to telomerase activity determined by the highly quantitative droplet digital TRAP assay [72, 73]. The role that telomere lengths have on the regulation of gene expression, including hTERT, across the lifespan (termed telomere position effect—over long distances) in various populations is also warranted for consideration [74, 75]. Such investigations within immune cells will serve to further elucidate the impact of hTERT-telomerase-telomere length dynamics on the sequela of age-related inflammatory disease pathology at the cellular level.

## Figures and Tables

**Table 1:** Subject anthropometric and cardiovascular measures.

Variable	Young adult (*n* = 15)	Middle aged (*n* = 15)	*p* value
Sex (M/F)	7/8	3/12	0.130
Age (y)	25.27 ± 3.26	57.27 ± 6.75	≤0.001^∗^
Weight (kg)	66.54 ± 13.39	66.91 ± 9.94	0.932
Height (m)	1.73 ± 0.11	1.68 ± 0.07	0.141
BMI (kg∙m^−2^)	22.00 ± 2.49	23.50 ± 2.03	0.081
Waist (cm)	73.65 ± 8.87	80.15 ± 7.51	0.039^∗^
Hip (cm)	99.84 ± 7.04	99.26 ± 5.95	0.809
W : H ratio	0.74 ± 0.06	0.81 ± 0.07	0.005^∗^
Body fat percentage (%)	19.41 ± 6.21	28.55 ± 8.43	0.005^∗^
Sagittal diameter (cm)	20.08 ± 2.22	23.01 ± 2.04	0.002^∗^
Resting HR (bpm)	67.07 ± 9.54	66.07 ± 12.66	0.809
Resting SBP (mmHg)	113.20 ± 9.375	120.13 ± 18.59	0.211
Resting DBP (mmHg)	74.40 ± 11.54	75.73 ± 10.14	0.739
MAP	87.33 ± 10.00	88.53 ± 13.25	0.782

∗ indicates a significant difference between middle-aged and young adults (*p* < 0.05). Data are presented as means ± S.D. BMI: body mass index; W : H: waist-to-hip ratio; HR: heart rate; SBP: systolic blood pressure; DBP: diastolic blood pressure; MAP: mean arterial pressure.

## Data Availability

The data used to support the findings of this study are available from the corresponding author upon request.
